# *In Vitro* Cytotoxicity of Calcium Silicate-Based Endodontic Cement as Root-End Filling Materials

**DOI:** 10.1155/2016/9203932

**Published:** 2016-01-21

**Authors:** Selen Küçükkaya, Mehmet Ömer Görduysus, Naciye Dilara Zeybek, Sevda Fatma Müftüoğlu

**Affiliations:** ^1^Department of Endodontics, Faculty of Dentistry, Hacettepe University, 06100 Ankara, Turkey; ^2^Department of Endodontics, Dental College, University of Sharjah, Sharjah, UAE; ^3^Department of Histology and Embryology, Faculty of Medicine, Hacettepe University, 06100 Ankara, Turkey

## Abstract

The aim of this study was to evaluate the cytotoxicity of three types of calcium silicate-based endodontic cement after different incubation periods with human periodontal ligament fibroblasts. Human periodontal ligament fibroblasts were cultured from extracted third molars and seeded in 96-well plates. MTA, calcium enriched mixture (CEM) cement, and Biodentine were prepared and added to culture insert plates which were immediately placed into 96-well plates containing cultured cells. After incubation periods of 24, 48, and 72 hours, cell viability was determined with WST-1 assay. Data were analysed statistically by ANOVA with repeated measures and Bonferroni tests. There was no significant difference in cell viability amongst the test materials after each incubation period (*P* > 0.05). MTA and CEM presented more than 90% cell viability after 24 and 48 hours of incubation and showed statistically significant decrease in cell viability after 72 hours of incubation (*P* < 0.05). Biodentine showed significantly less cell viability (73%) after 24 hours of incubation, whereas more than 90% cell viability was seen after 48 and 72 hours of incubation (*P* < 0.05). Despite the significant changes in cell viability over time, materials presented similar cytotoxicity profile. Biodentine and CEM can be considered as alternative materials for root-end surgery procedures.

## 1. Introduction

Root-end surgery is a viable treatment option in the presence of persistent periradicular pathosis or when orthograde retreatment is considered unfeasible [1]. The ultimate success of the root-end surgery depends on the regeneration of a functional periodontal attachment apparatus, including cementum overlying the resected root-end surface, periodontal ligament (PDL), and alveolar bone [2]. To achieve this goal, it has been suggested to place a root-end filling material that not only prevents egress of any remaining bacteria or their by-products but also allows for the formation of a normal periodontium across its surface [3].

An ideal root-end filling material should be biocompatible, insoluble, dimensionally stable, radiopaque, antibacterial, and easy to manipulate and have effective sealing ability [4]. Because existing materials did not fulfill these characteristics, mineral trioxide aggregate (MTA), a calcium silicate-based material, was developed [5] and recommended for root-end filling because of its good physical and chemical properties [6]. MTA appears to be the most promising material to date, as it comes closest to being the ideal material for root-end filling. Nevertheless, MTA has some drawbacks such as a long setting time, difficult handling characteristics, and presence of toxic elements in the material composition [7]. Recently, new materials have been developed to be used for similar indications to MTA. Biodentine (Septodont, SaintMaur-des-Fosses, France), a more recent calcium silicate-based material, was introduced as a dentin substitute under resin composite restorations. Biodentine contains tricalcium silicate, calcium carbonate, and zirconium oxide and a water-based liquid-containing calcium chloride as the setting accelerator and a water-reducing agent [8]. Biodentine has been reported to exhibit short setting time and high mechanical properties [9]. Calcium enriched mixture (CEM cement, BioniqueDent, Tehran, Iran) is a recently introduced endodontic material which consists of several calcium compounds such as calcium silicate, calcium oxide, calcium phosphate, calcium carbonate, calcium sulfate, and calcium chloride [10]. It has been reported to have good handling characteristics, an ability to form hydroxyapatite in contact with tissue fluid [11], and superior antibacterial properties to that of MTA [12].

Biocompatibility is an important quality of root-end filling materials and cytotoxicity tests are primary biocompatibility tests which measures the capacity of a material to impact on cellular viability. Limited comparative data exist about the cytotoxicity of MTA, CEM, and Biodentine [13]. This study aimed to assess the cytotoxicity of MTA, CEM, and Biodentine on cultured human periodontal ligament fibroblasts using WST-1 (4-[3-(4-iodophenyl)-2-(4-nitrophenyl)-2H-5-tetrazolio]-1,3-benzene disulfonate) assay.

## 2. Materials and Methods

### 2.1. Cell Culture Preparation

**Figure 1:**
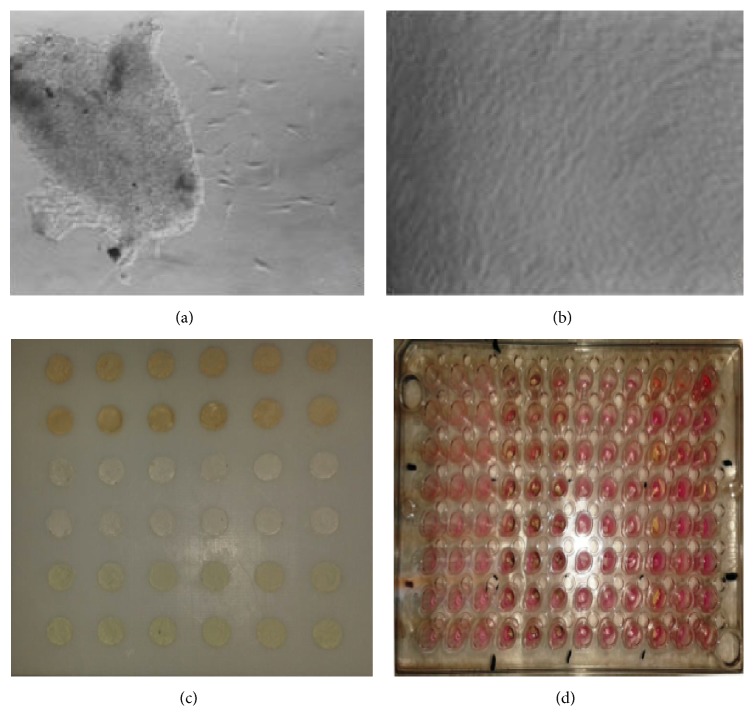
(a) Outgrowth of fibroblast cells from the tissue section (×40 magnification). (b) Fibroblast cells reached confluency (×40 magnification). (c) Materials inserted into cylindrical polyethylene molds. (d) The culture insert plate with materials placed into the 96-well plate.

Following the university ethics committee approval (Ethics Board number GO-13/545), human periodontal tissue was obtained from extracted third molars of patients who had given their informed consent. The periodontal tissue was removed from the roots of the teeth and then divided into small pieces with sterile instruments. Periodontal tissue specimens were placed into 25 cm^2^ tissue culture flasks and were incubated with Dulbecco's modified Eagle medium (DMEM; Hyclone, Thermo Scientific, Logan, UT, USA) containing 10% foetal bovine serum (FBS, Hyclone), 10000 units/mL penicillin, 10 mg/mL streptomycin, and 200 mM L-glutamine. The flasks were maintained at 37°C in a humidified incubator in an atmosphere of 5% CO_2_. The medium was refreshed every 2 days. When outgrowth of cells was observed under phase contrast microscope (Figure 1(a)), the medium was replaced twice weekly until cells reached 75% confluence (Figure 1(b)). The PDL fibroblasts from the fourth passage were used for the experiments. Prior to experimental tests, the cells were seeded at 1 × 10^4^ cells/well in 96-well plates and 100 *μ*L medium was added to each well and incubated for 24 hours in an atmosphere of 5% CO_2_ at 37°C.

### 2.2. Preparation of Test Materials

The following materials were tested: White MTA Angelus (Angelus, Londrina, PR, Brazil), CEM (BioniqueDent, Tehran, Iran), and Biodentine (Septodont, Saint Maur des Fossés, France). Nine samples of each material were prepared according to the manufacturer's instructions and inserted into cylindrical polyethylene molds measuring 5 mm wide and 2 mm high (Figure 1(c)). Samples were stored at 37°C in a chamber of 100% relative humidity before sterilization with ultraviolet rays for 24 hours. Thereafter, materials were added to culture insert plates (Millicell-96 Cell Culture Insert Plate, PSHT004S5, Millipore, Darmstadt, Germany), which were immediately placed into 96-well plates containing the culture medium and cells (Figure 1(d)). Cells cultured with only culture insert plates without test materials served as controls. After incubation periods of 24, 48, and 72 hours, each culture insert plate was removed and 96-well plates were used for cytotoxicity assay.

### 2.3. Cytotoxicity Assay

The Cell Proliferation Reagent WST-1 (Roche Diagnostics, Mannheim, Germany) was used to assess cell viability. This assay is based on the cleavage of a tetrazolium salt (WST-1) to soluble formazan dye by the mitochondrial dehydrogenase of living cells. At indicated time-points, 10 *μ*L of the WST-1 reagent was added to each well, and the plates were incubated at 37°C for 4 h. Subsequently, the optical densities of the plates were detected at 440 nm by micro-ELISA Reader (Versamax microplate reader, Molecular Devices). The percentage of relative cell viability was calculated using the following formula: (Test optical density/control optical density) *∗* 100. All data were entered into the Statistical Package for the Social Sciences program (SPSS for Windows 11.0; SPSS Inc., Chicago, IL). All results were analysed statistically by ANOVA with repeated measures and Bonferroni tests.

## 3. Results

**Figure 2:**
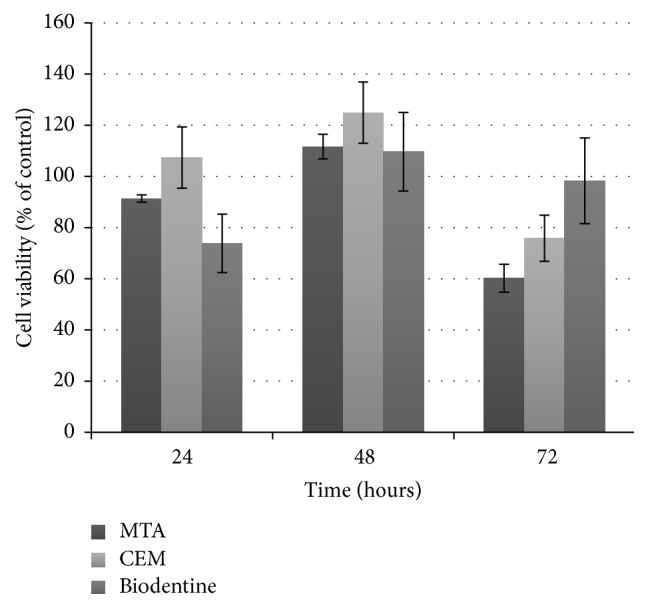
Cell viability of cultured human periodontal ligament fibroblasts after incubation with the tested materials for 24, 48, and 72 hours. Each bar represents the mean absorbance ± standard deviation.

The cell viability of human PDL fibroblasts according to the groups is presented in Figure 2. There was no significant difference in cell viability between the test materials after 24, 48, and 72 hours of incubation (*P* > 0.05). However, significant changes were seen in cell viability over time for each material. MTA and CEM showed more than 90% cell viability after 24 and 48 hours of incubation, while they presented significantly less cell viability after 72 hours of incubation (60% and 75% cell viability, resp.) (*P* < 0.05). In contrast, Biodentine showed significantly less cell viability (73% cell viability) after 24 hours of incubation, whereas more than 90% cell viability was seen after 48 and 72 hours of incubation (*P* < 0.05).

## 4. Discussion

There are several important issues to consider in the experimental design of* in vitro* cytotoxicity studies such as the choice of appropriate cell type, passage number, and assay type [3]. In this study, human PDL fibroblasts were used to simulate the clinical environment. The healing after root-end surgery includes the regrowth of PDL along the resected root surface [3]. Therefore, it is important to test how root-end filling materials affect the PDL fibroblasts. Cell lines with high passage numbers exhibit alterations in cell morphology [14]. In this study, we used fourth passage of cell lines which, being younger passages, presented minimal cell changes due to cell culture manipulation [14]. It has been proposed that cytotoxicity assay type should depend on the chemical composition of the test materials [3]. The tested materials in the present study are hydrophilic substances likely to release ionic components and interfere with intracellular enzyme activities. Thus, it is logical to choose an assay which measures mitochondrial dehydrogenase activity. The most widely used assay for this purpose is MTT assay and it is a first-generation tetrazolium derivative which is reduced in metabolically active cells by a mitochondrial enzyme. However, MTT assay forms insoluble formazan crystals that requires the addition of a detergent and this step can complicate the assay [15]. WST-1 is a second-generation tetrazolium derivative which works similarly to MTT assay by reacting with the mitochondrial succinate tetrazolium reductase. WST-1 is metabolized into nontoxic, water-soluble, membrane-permeant products and does not require the solubilization step [15]. Therefore, WST-1 assay was used to determine the cell viability in the present study.

Adequate contact between cells and test material is also crucial to cell cytotoxicity testing. Contact between cells and material can be achieved in various ways [16]. Direct cell-material contact is a clinically relevant* in vitro* test model considering the exposure pattern between the root-end filling materials and periapical tissues. On the other hand, direct cell-material contact can influence cell viability through physical factors. It has been shown that the effect of direct contact between the cells and the material on testing may decrease the sensitivity of an* in vitro *system [17]. Besides, the particulate and solid nature of the test materials may interfere with reading in the optical reader when the materials are placed in direct contact with cells. At this point using material extracts could be a viable solution to assess the cytotoxicity indirectly. However, it would be less clinically relevant considering the limited solubility capacity of the test materials and their ability to release different molecules through time due to their setting reactions and interaction with the cells [18]. To bypass these problems, we used culture insert plates to carry the test materials through incubation periods. Cells come into close proximity to the test materials while soluble compounds from the materials reach the cells through the pores of culture insert plates [17]. Therefore, using culture insert plates allowed repeated and consecutive exposure of the same cells over an extended time.

In the present study, no significant difference was detected between MTA, CEM, and Biodentine for each incubation period. These results are in agreement with those found in previous cytotoxicity studies [19–23]. However, cell viability showed significant differences over time for all materials. MTA and CEM showed a statistically significant decrease in cell viability after 72 hours of incubation whereas Biodentine showed less cell viability after 24 hours of incubation compared with other time periods. The reason for the decrease in cell viability for MTA and CEM after 72 hours of incubation may be the production of calcium hydroxide due to the hydration reaction in the materials [10, 24]. Similarly, calcium hydroxide is produced as a by-product of the reaction in Biodentine [25]. However, the liquid for mixing with the Biodentine powder consists of calcium chloride which results in accelerated cement by decreasing the setting time [25]. Early production of calcium hydroxide can explain why Biodentine showed relatively less cell viability after 24 hours of incubation rather than other time periods [26]. It is also important to consider the clinical significance of this issue. The gradual release of hydroxyl ions may decrease cell viability* in vitro*. However, under* in vivo* conditions high alkalinity due to hydroxyl ions may be neutralized by the body tissue fluid and may not cause significant effects on cell viability [20].

Degradation products and elution substances from materials can induce cytotoxicity [27]. In this regard, there have been some concerns about the purity of MTA since it is based on a clinker related to Portland cement. According to recent studies, MTA showed evidence of heavy metals such as arsenic, chromium, and lead and the presence of aluminate phase which have been associated with toxicity [28, 29]. Biodentine is claimed by the manufacturer as a high purity dental material due to its production with Active Biosilicate Technology. However, a recent study has found traces of arsenic, chromium, and lead in elutes from a mixed Biodentine solution but also verified that aluminate phase is not included in Biodentine [30]. The biocompatibility of radiopacifiers in materials can be another important issue since they have been observed in high levels in tissues adjacent to the material [31]. MTA has bismuth oxide as a radiopacifier [6]. Several studies demonstrated that the addition of bismuth oxide to Portland cement showed higher cytotoxicity compared with Portland cement alone [32, 33]. On the other hand, Biodentine contains zirconium oxide as a radiopacifier which presents a lower toxicity profile than bismuth oxide [27, 34]. These properties may have contributed to the results of Biodentine which demonstrated relatively high cell viability at all time periods in the present study.

Laboratory studies cannot simulate the complex biological conditions of the clinical status. Therefore, the results obtained from the preliminary cytotoxicity tests have limitations with respect to direct correlation with clinical situations. However, they do provide reproducible and reliable means for comparing and testing new materials and establishing international standards. According to the results of the present* in vitro* study all materials showed changes in cell viability over time; however, there was no difference between materials in terms of cytotoxicity and each material presented acceptable cell viability as being consistent with a limited number of* in vivo* studies in the literature [35–37].

In conclusion, CEM and Biodentine presented similar cytotoxicity profiles to MTA and can be considered as alternative materials for root-end surgery procedures.
